# Age-Related Macular Degeneration: A Scientometric Analysis

**Published:** 2015

**Authors:** Shahrokh Ramin, Masoud Soheilian, Gholamreza Habibi, Roghayeh Ghazavi, Reza Gharebaghi, Fatemeh Heidary

**Affiliations:** 1 Ophthalmic Research Center, Shahid Beheshti University of Medical Sciences, Tehran, Iran; 2 Farzan Scientometric Group, Farzan Clinical Research Institute, Tehran, Iran; 3 Health Policy Research Center, Shiraz University of Medical Sciences, Shiraz, Iran

**Keywords:** Age-related Macular Degeneration, Bibliometrics, Historiography, Scientometrics, Citation Analysis

## Abstract

Age-related macular degeneration (ARMD) is a major cause of central blindness among working aged adults across the world. Systematic research planning on any subject, including ARMD is in need of solid data regarding previous efforts in this field and to identify the gaps in the research. This study aimed to elucidate the most important trends, directions, and gap in this subject.

The data extracted from the Institute for Scientific Information were used to perform a bibliometric analysis of the scientific productions (1993–2013) about ARMD. Specific parameters related to ARMD were analyzed to obtain a view of the topic’s structure, history, and document relationships. Additionally, the trends and authors in the most influential publications were analyzed.

The number of articles in this field was found constantly increasing. Most highly cited articles addressed genetic epidemiology and clinical research topics in this field. During the past 3 years, there has been a trend toward biomarker research.

Through performing the first scientometric survey on ARMD research, we analyzed the characteristics of papers and the trends in scientific production. We also identified some of the critical gaps in the current research efforts that would help in large-scale research strategic planning.

## INTRODUCTION

Age-related macular degeneration (ARMD) is one of the top four causes of blindness in elderly people. ARMD, described more than 80 years ago, is a progressive disease of the central area in the ocular posterior segment (the macula lutea), which leads to a gradual deterioration in central vision and causes severe disability to affected individuals. In North America, Europe, and Australia, ARMD accounts for up to half of all cases of central blindness, affecting approximately 3% of the general adult population. In the United States, about 12%–15% of people older than 80 years of age were estimated to have ARMD in 2000, and this number is expected to be more than 2.95 million in 2020. People with ARMD have been found to experience reduced quality of life, depression, and difficulty with the activities of daily living, which pose serious financial burden on their family in terms of high medical and societal costs that are due to increased risk of falling, need for vision enhancing equipment, depression/anxiety treatment, and assistance with activities of daily living (1, 2).

Visual loss in late ARMD can be caused by either of the following two processes that cause photoreceptor dysfunction: geographic atrophy (GA) or choroidal neovascularization. GA refers to the confluent atrophy of the choriocapillaris and associated retinal pigment epithelium (RPE). The RPE is the outermost layer of the retina, which is involved in phagocytosis of the photoreceptor outer segments and biologic maintenance. In choroidal neovascularization, an ingrowth of new vessels occurs from the choriocapillaris invading the retina. These new vessels leak serous fluids beneath and into the neural retina causing fibrous scarring, which defines the late stage of ARMD (exudative, or neovascular) (3). Non-exudative (dry) ARMD is often marked by the formation of drusen, pigmentary changes in the RPE, and atrophy of the RPE. Dry ARMD is more common; in one series of autopsy eyes, ARMD was found in 33% of patients older than 65 years (4, 5). 

The incidence of the disease increases with age. Through major breakthrough discoveries made in the last decade in treating the wet form of ARMD, the chance of stabilizing or increasing vision has been increased to 90%. Nonetheless, this improvement is associated with a significant price tag of monthly intravitreal injections (with the ever-present phantom of endophthalmitis and other injection-related adverse events) and uncertainty regarding the duration of treatment (6). 

A scientometrics method is one that measures and analyzes scientific publications related to a specific topic regarding the trends in citations, most important content, authors, and journals. A widespread use of scientometric method goes back to 1960s when Eugene Garfield finalized the construction of Science (7). In this article, we performed the first scientometric analysis of the ARMD field to elucidate the most important trends and directions of this subject.

## MATERIAL AND METHODS

A bibliometric study was performed on the articles related to “Age-Related Macular Degeneration” published between 1993 and 2013. The Institute for Scientific Information (ISI) web of science available at http://www.isiknowledge.com was our main source. Two mesh terms—“Age-Related Macular Degeneration” and “ARMD”—already checked in Pubmed mesh database were used to conduct the search.

Only original articles were selected for further evaluation. These articles were evaluated regarding citation characteristics, contributing role of each author, country, funding agency, institution, journal, and language of published articles. Articles were also evaluated regarding the trend of publication and citation during a selected time and also subject areas covered. Articles that were published in 10 countries with the greatest number of publications on the topic were analyzed separately.

Articles published by each country were evaluated regarding subject areas and publishing journals. Special attention was paid to total citations with and without self-citation, citation per year, and citation per item for journals of each country. Articles that were cited more than 100 times were evaluated regarding year of publication, country of affiliation of the first author, and publishing journal. Number, country, and year of collaborative studies were also considered. All three resources available in the ISI web of science were used for this purpose (Science Citation Index Expanded, Social Sciences Citation Index, The Arts & Humanities Citation Index). For the citation analysis, two parameters were calculated: Local Citation Score (LCS) and Global Citation Score (GCS). LCS listed all papers sorted by citation frequency within the local (the starting bibliography) collection; however, GCS counted citations in the whole collection. For the citation burst analysis, first, 100 key words that generated the citation bursts were extracted, and then non-specific and general key words were omitted.

## RESULTS

*Annual Publication Number During 1993-2013*

**Figure 1:**
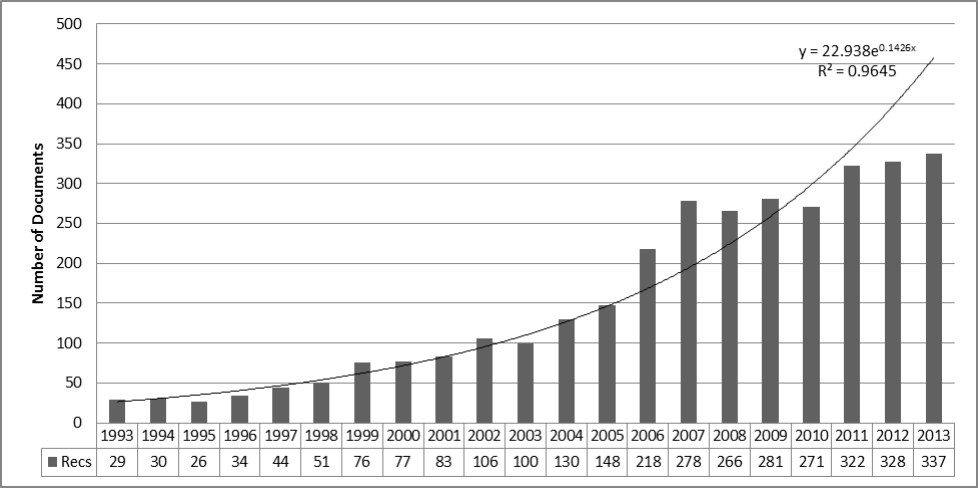
Annual Number of Published Papers

A total of 3235 research articles were available on ARMD in the ISI Web of Science during 1993-2013. These papers were drafted by 10,706 authors, 2332 institutions, and 67 countries and were published in 388 journals in 9 languages. Figure 1 demonstrates the growth rate of publications in this field (14.46% per year). The H-index of this subject was 125.

*Citation Profile of Articles*

The total LCS citations were 29,924 in number and GCS citations were 91,840 in number. The average citation per paper (C/P) was 28.39

**Figure 2:**
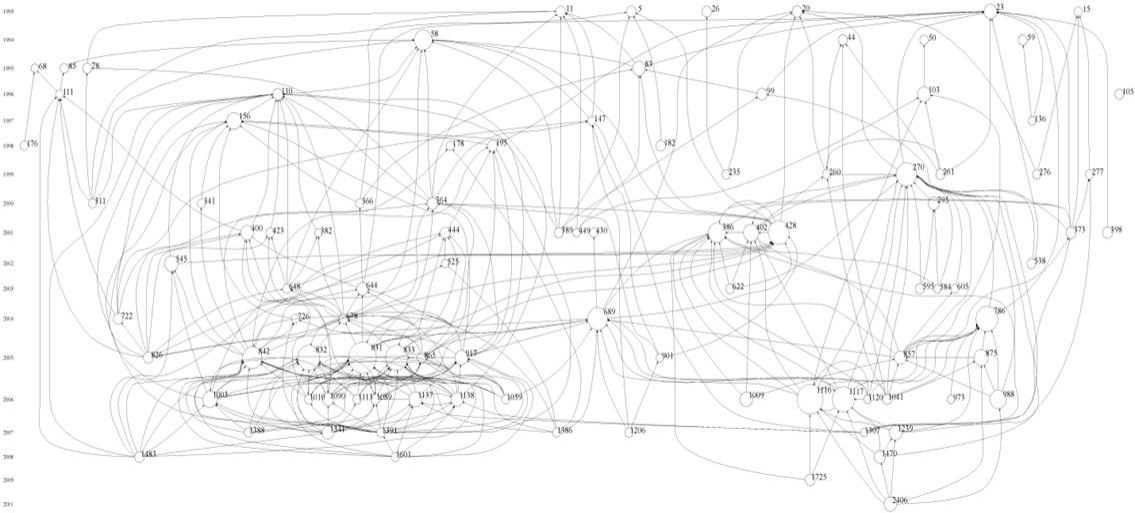
Histogram Map of 20 Years of Research in ARMD

Table 1 shows the articles that were cited 100 or more times. Figure 2 shows the trend of citations during the period. Appendix 1 shows the highly cited articles in this field. Figure 1 shows the histogram map of 20 years of research in this field.

**Figure 3:**
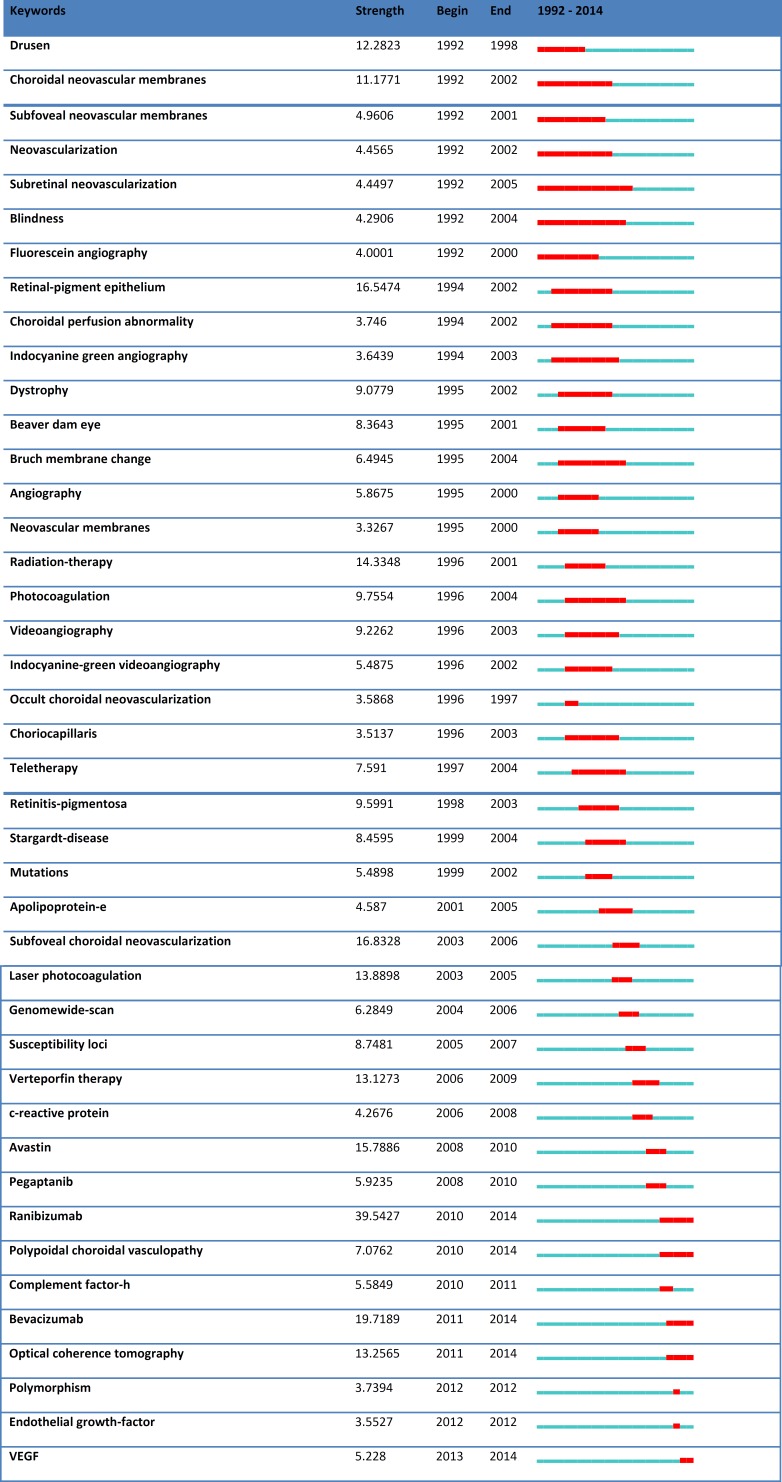
Citation Burst Analysis

Figure 3 shows the key words generating the highest citation bursts and the time periods associated with them. The key words associated with the highest citation bursts included: drusen, choroidal neovascular membranes, subfoveal neovascular membranes, neovascularization, subretinal neovascularization, blindness, fluorescein angiography, retinal pigment epithelium, choroidal perfusion abnormality, indocyanine green angiography, dystrophy, Beaver dam eye, Bruch membrane change, angiography, neovascular membranes, radiation therapy, photocoagulation, videoangiography, indocyanine green videoangiography, occult choroidal neovascularization, choriocapillaris, teletherapy, retinitis pigmentosa, Stargardt disease, mutations, apolipoprotein E, subfoveal choroidal neovascularization, laser photocoagulation, genome-wide scan, susceptibility loci, verteporfin therapy, c-reactive protein, avastin, pegaptanib, ranibizumab, polypoidal choroidal vasculopathy, complement factor H, bevacizumab, optical coherence tomography, polymorphism, endothelial growth factor, and vascular endothelial growth factor.

*Subject Analysis of the Most Highly Cited Documents*

The most common topics of the top 10 highly cited papers were genetic epidemiology research (40%), clinical study (surgical or pharmaceutical treatment) (50%), and epidemiologic study (10%) (Table 1). *Languages, Journal Subjects, and Author Profiles of Publications *

Most ARMD articles were in English (3068), followed by German (105) and French (47). In total, the articles were written in nine languages (English, German, French, Portuguese, Hungarian, Spanish, Polish, Serbian, and Slovene). Dr R. Klein, with 80 articles, had the largest number of publications in the field of ARMD research (Table 2). When analyzed based on the number of papers in ARMD, 8 out of top 10 journals were general medical journals (such as the New England Journal of Medicine) and the remaining were ophthalmology journals. But when the same calculation was made based on the citation number (TLCS), seven journals were general medical journals and three were ophthalmology journals. When analyzed based on TGCS, highly cited papers were mostly published in general medical journals (80%), and the remaining 20% were published in Ophthalmology journals (Tables 3). No correlation was found between the impact factor of the most highly influential journals in this field and the total citations they had received for their papers in the field of ARMD.

A majority of the top 10 universities and institutions in the list are from the United States and Australia. The first two of them are the Johns Hopkins University and University of Melbourne in terms of number of documents and Harvard University and University of Wisconsin in terms of number of citations (Table 4).

*Geographical Distribution*

In general, 67 countries contributed to the promotion of the field of ARMD by publishing articles. The United States, Germany, and the UK had the highest number of documents but they had the highest number of citations to their research papers in the field of ARMD (Table 5). 

**Table 1:** Articles With Highest Number of Citations

**#**	**Author/ Title / Journal**		**CITATION**
**1**	Klein Rj, Zeiss C, Chew Ey, Tsai Jy, Sackler Rs, Et Al. Complement Factor H Polymorphism In Age-Related Macular Degeneration Science. 2005 Apr 15; 308 (5720): 385-389		1814
**2**	Rosenfeld Pj, Brown Dm, Heier Js, Boyer Ds, Kaiser Pk, Et Al. Ranibizumab For Neovascular Age-Related Macular Degeneration New England Journal Of Medicine. 2006 Oct 5; 355 (14): 1419-1431		1662
**3**	Edwards Ao, Ritter R, Abel Kj, Manning A, Panhuysen C, Et Al. Complement Factor H Polymorphism And Age-Related Macular Degeneration Science. 2005 Apr 15; 308 (5720): 421-424		1173
**4**	Brown Dm, Kaiser Pk, Michels M, Soubrane G, Heier Js, Et Al. Ranibizumab Versus Verteporfin For Neovascular Age-Related Macular Degeneration New England Journal Of Medicine. 2006 Oct 5; 355 (14): 1432-1444		1161
**5**	Haines Jl, Hauser Ma, Schmidt S, Scott Wk, Olson Lm, Et Al. Complement Factor H Variant Increases The Risk Of Age-Related Macular Degeneration Science. 2005 Apr 15; 308 (5720): 419-421		1148
**6**	Gragoudas Es, Adamis Ap, Cunningham Et, Feinsod M, Guyer Dr Pegaptanib For Neovascular Age-Related Macular Degeneration New England Journal Of Medicine. 2004 Dec 30; 351 (27): 2805-2816		1146
**7**	Kassoff A, Kassoff J, Buehler J, Eglow M, Kaufman F, Et Al. A Randomized, Placebo-Controlled, Clinical Trial of High-Dose Supplementation With Vitamins C And E, Beta Carotene, And Zinc For Age-Related Macular Degeneration And Vision Loss - Areds Report No. 8 Archives Of Ophthalmology. 2001 Oct; 119 (10): 1417-1436		1081
**8**	Bressler Nm Photodynamic Therapy Of Subfoveal Choroidal Neovascularization In Age-Related Macular Degeneration With Verteporfin - One-Year Results Of 2 Randomized Clinical Trials - Tap Report 1 Archives Of Ophthalmology. 1999 Oct; 117 (10): 1329-1345		1021
**9**	Friedman Ds, O'colmain B, Tomany Sc, Mccarty C, De Jong Ptvm, Et Al. Prevalence Of Age-Related Macular Degeneration In The United States Archives Of Ophthalmology. 2004 Apr; 122 (4): 564-572		970
**10**	Hageman Gs, Anderson Dh, Johnson Lv, Hancox Ls, Taiber Aj, Et Al. A Common Haplotype In The Complement Regulatory Gene Factor H (Hf1/Cfh) Predisposes Individuals To Age-Related Macular Degeneration Proceedings Of The National Academy Of Sciences Of The United States Of America. 2005 May 17; 102 (20): 7227-7232		929

**Table 2:** Most Active Authors in the Field of ARMD Research

**#**	**Author**	**Recs**	**TLCS**	**TGCS**
**1**	Klein R	80	2405	6370
**2**	Bressler Nm	76	3639	10146
**3**	Mitchell P	63	1300	3350
**4**	Soubrane G	60	1202	3152
**5**	Seddon Jm	58	2269	6377
**6**	Schmidt-Erfurth U	56	1511	4504
**7**	Holz Fg	55	550	2187
**8**	Guymer Rh	52	310	855
**9**	Wong Ty	52	422	1004
**10**	Bressler Sb	48	2546	6735

The growth rate of 14.6% in the article numbers was observed.

**Table 3:** Journals with Highest Number of Papers in This Field

**#**	**Journal**	**Records**	**Citation **	**2 year IF**	**5 year IF**
**1**	Ophthalmology	289	12624	5.563	5.777
**2**	Investigative Ophthalmology & Visual Science	274	7421	3.441	3.730
**3**	American Journal Of Ophthalmology	232	8043	3.631	4.292
**4**	Retina-The Journal Of Retinal And Vitreous Diseases	225	3933	2.825	2.761
**5**	British Journal Of Ophthalmology	198	4462	2.725	3.023
**6**	Archives Of Ophthalmology	166	13369	3.826	4.160
**7**	Graefes Archive For Clinical And Experimental Ophthalmology	125	2550	1.932	2.037
**8**	Eye	97	1015	1.818	1.883
**9**	Molecular Vision	73	1210	1.987	2.311
**10**	Acta Ophthalmologica	65	408	2.345	2.428
**11**	Ophthalmologica	57	503	1.412	1.236
**12**	Klinische Monatsblatter Fur Augenheilkunde	49	197	0.699	0.473
**13**	Ophthalmologe	48	295	0.529	0.681
**14**	European Journal Of Ophthalmology	46	296	0.912	0.963
**15**	Journal Francais D Ophtalmologie	45	112	0.438	0.443
**16**	Japanese Journal Of Ophthalmology	43	234	1.274	1.488
**17**	PLose One	43	493	3.730	4.244
**18**	Ophthalmic Surgery Lasers & Imaging	39	634	1.464	0.922
**19**	Canadian Journal Of Ophthalmology-Journal Canadien D Ophtalmologie	38	398	1.145	1.320
**20**	Clinical And Experimental Ophthalmology	28	291	1.964	2.047

**Table 4:** Institutions with Highest Number of Papers

#	**Institution**	**Records**	**Citations **
**1**	Johns Hopkins Univ	130	7472
**2**	Univ Melbourne	125	3103
**3**	Harvard Univ	117	9211
**4**	Duke Univ	97	4450
**5**	Univ Wisconsin	97	6462
**6**	Nei	86	6282
**7**	Univ Sydney	79	3584
**8**	Moorfields Eye Hosp	73	2029
**9**	Univ Penn	73	3241
**10**	Univ Southern Calif	68	1737
**11**	Univ Heidelberg	64	1960
**12**	Univ Miami	57	5242
**13**	UCL	54	1273
**14**	Univ Paris 12	53	2140
**15**	Natl Univ Singapore	51	809
**16**	Univ Cologne	50	1054
**17**	Univ Utah	49	2540
**19**	Univ Iowa	48	3215
**20**	Univ Bonn	47	1190

## Discussion

We analyzed the subject of highly cited papers, dividing them into two broad categories: epidemiology versus clinical research and translational versus basic science research (Appendix 1). Most of the highly cited papers were genetic epidemiology or clinical reports. Although ARMD is a debilitating disease and adversely affects the quality of life and emotional status of subjects, highly cited papers had largely neglected this subject. Highly cited reports also addressed the following subjects more frequently: (1) association of various genotypes with ARMD, (2) chemotherapy for wet ARMD, including the intravitreal treatment, and (3) effect of diet and vitamins on ARMD. Recently, as Appendix 2 shows, there has been a trend toward more applicable genetic epidemiology and translational research (biomarkers). In cluster analysis for the citations, we found the following three major clusters in the citation histogram map (Figure 1): (1) complement H factor polymorphism in the ARMD (nodes 831, 832, 833, and 842 in the index 1), (2) treatment of subfoveal choroidal neovascularization in ARMD (nodes 386, 402, and 428), and (3) ranibizumab treatment for neovascular ARMD (nodes 1116 and 1117). 

**Table 5:** Countries With Highest Number of Papers in the Field of ARMD

**#**	**Country**	**Recs**	**CITATION **
**1**	USA	1338	60915
**2**	Germany	363	8968
**3**	UK	340	9990
**4**	Australia	215	6031
**5**	Japan	193	3740
**6**	France	163	4862
**7**	Peoples R China	144	2486
**8**	Italy	120	1842
**9**	Austria	112	3371
**10**	Unknown	105	2707
**11**	Canada	97	3205
**12**	Switzerland	92	2959
**13**	Netherlands	74	4699
**14**	Spain	65	1184

 Proportionally, articles related to medical retina compared to other subspecialties have increased between 2005 and 2009. In an analytical study of the ophthalmology research papers, case–control or cohort studies comprised most study designs used (40.1%), followed by non-analytic studies (28.7%), basic science (24.6%), RCTs (3.3%), review articles (2.6%), and meta-analyses (0.3%) (8). However, the trend was not similar in the ARMD research for the highly cited papers. As Graph 3 shows, in the 1990s, the key words that resulted in the most strong citation bursts were the ones associated with pathophysiology, such as subretinal neovascularization; however, in the past 4 years, there has been a trend toward novel treatments such as ranibizumab in the context of RCTs, which shows the importance of this line of research and also the progression in the field of ARMD. Both the citation burst analysis and the histography of the most cited papers in the past 3 years showed that genetic epidemiology topics are among the recent hot topics in this field. Interestingly, genetic epidemiology studies also comprised the most highly cited articles in the past 20 years, which implies that this field is still hot and many more studies have a high chance of publication in this field.In our study, the number of citations correlated poorly with the impact factor of the journals. Citation frequency and impact factor both render important information regarding the merit of a paper, but the ranking of research groups on the basis of journal impact factor is shown to have little correlation with the ranking of the same groups on the basis of citation frequency. This can be due to the fact that journals with an advance online publication had higher impact factors compared with those without an advance online publication. Also, researchers might prefer to publish their results in their subspecialty journal, which might not necessarily have a high impact factor. It is suggested that “citation analysis is a good rough indicator of the quality of work as it is perceived by other scientists” (9). Previously, two detailed citation analysis reports have been published, one spanning the period of 1850–1949 (10) and the other 1975–2006 (11). In both reports, ARMD was a major citation classic, especially for ophthalmology journals. The second report surveyed 46 ophthalmology journals and concluded that the 100 most cited articles were published in 13 journals, including the Archives of Ophthalmology, Ophthalmology, and the American Journal of Ophthalmology (11). Epidemiology of ARMD was one of the major topics of 100 most cited articles. Also we found that the H-index of ARMD was 125, which indicates the appreciation of the context of ARMD within vision research. The publications of Dr Klein and several other top researchers in the field of ARMD research are also among the top 100 most cited articles in the field of Ophthalmology, which shows the importance of this field.

Several lines of research have been previously identified as priorities in ARMD research. These include temporal patterns and changing the prevalence of ARMD; improvements of ocular imaging (eg, high-resolution OCT) to allow better phenotype classification of both early and late ARMD; and epidemiologic studies to determine gene-environment interactions for ARMD to identify early modifiable risk factors to prevent ARMD (12). According to the results of our study, several of these areas do not receive enough attention from the experts in the field, and better research strategies should be implemented.

In conclusion, the result of our report as the first scientometric analysis of the research on ARMD can be used as a guideline for authors, researchers, and policy makers to determine the best ways to allocate their financial and workforce resources.
